# Tackling cardiometabolic disease in southeast Asia: local research to inform local practice

**DOI:** 10.1016/j.lansea.2024.100491

**Published:** 2024-10-10

**Authors:** The Lancet Regional Health – Southeast Asia

Cardiometabolic disease is a term used to indicate not only cardiovascular but also metabolic disorders such as diabetes. Although mortality due to cardiovascular disease in high-income countries (HICs) seems to be declining, it is rising in low-income and middle-income countries (LMICs), with higher mortality among people from low socioeconomic backgrounds and younger age groups. Cardiometabolic diseases are linked to several risk factors, in particular obesity, hypertension, diet, tobacco, air pollution, and physical inactivity. Even though the overall risk factor burden appears to be lower in LMICs than in HICs, rates of major cardiovascular events, such as death from cardiovascular causes, myocardial infarction, stroke, or heart failure, are lower in HICs than in LMICs. Non-communicable diseases (NCDs), which are the main drivers of cardiometabolic disease, caused nearly two-thirds of all deaths in countries of the WHO South-East Asia Region in 2021, with half of these deaths in the age group 30–69 years; cardiovascular diseases (3.9 million), accounted for the most NCD deaths. With such a high burden of mortality, it is imperative that countries embrace innovative approaches to control the rising burden and strengthen health systems, in order to reduce the preventable mortality from these diseases.

Effective control and management of cardiometabolic disease in the region is challenged by limited resources, high out-of-pocket expenditure, lack of local guidelines, low health literacy, and political reluctance. Lack of local research often leads to translation of knowledge gathered from HICs into practice and direct import of guidelines without any regard for different characteristics or disease presentation in local populations. Efforts to tackle this burden must emphasise effective control strategies, tailored for respective needs in each country and setting, informed by research conducted in the local context. Researchers are now trying to introduce socially and culturally accepted interventions to improve risk factor control in populations with a relatively high risk of cardiometabolic disease.

In this issue of *The Lancet Regional Health – Southeast Asia*, we present locally conducted research that has potential implications for evidence-based practice in the region. Researchers have explored interventions that aim at engaging communities for hypertension control through collective decision making and monitoring. Bhattarai and colleagues present findings from an intervention in Nepal, using community health workers to help people with hypertension to manage and monitor their blood pressure through lifestyle modification and adherence to antihypertensives. The mean systolic blood pressure decreased from 133.4 to 130.8 mm Hg (decrease of 2.6 mm Hg) in the control arm, and from 134.2 to 129.8 mm Hg (decrease of 4.4 mm Hg) in the intervention arm. Their findings provide encouraging results for researchers looking for contextual evidence in implementing interventions targeting individual, community, and health system barriers in low-resource settings.

Researchers are also attempting to introduce non-pharmacological interventions that are affordable, accessible, acceptable among local populations, and are considered more sustainable to modify lifestyle in people living with chronic conditions such as diabetes. Lean and colleagues demonstrate the results of traditional diet in Nepal people with type 2 diabetes of under 5 years known duration. The study demonstrated consistent metabolic improvements from the diet intervention, and a remission rate among those with data of 39% at 24 weeks, 29% at 52 weeks. This study was based in a routine care real-life setting, in a clinical population in Nepal with a loss to follow-up of 36% over 12 months; this limitation should be considered when designing future studies.

Another study from Thailand attempts to identify dietary changes, and to determine correlations between these changes and NCD cases during 1960–2020. In this longitudinal ecological study, Beckmann and colleagues conclude that consumption of unhealthy or unsustainable food groups have increased concurrently with NCD cases and environmental impacts over the last decades in Thailand.

These studies are shining examples of how well conducted research in low-resource settings may help in implementing evidence-based practice to reduce the burden of cardiometabolic disease, which continues to inflict huge losses to the health and economies of nations. There is a need to expand research focused on epidemiology (for a better understanding of disease pattens and trends in the population), interventions at the community level, impact evaluation of national programmes, and cost-effectiveness of cheaper alternatives to current practices. We also need more research to inform policy at the national level, such as the one in Thailand, to inform governments in creating more sustainable and healthier food systems and built environments. The research needs to inform which population groups and settings should be targeted and where resources need to be channelled. Innovations are key to tackle high burden of these disease with limited resources and cultural and socioeconomic challenges.

We at *The Lancet Regional Health – Southeast Asia* are keen to publish research that has the potential to impact millions of lives through effective interventions which are easy to implement, acceptable, affordable, and support engagement and empowerment of communities and individuals and enhance patient voices in decision making.

